# Stabilizing multicellularity through ratcheting

**DOI:** 10.1098/rstb.2015.0444

**Published:** 2016-08-19

**Authors:** Eric Libby, Peter L. Conlin, Ben Kerr, William C. Ratcliff

**Affiliations:** 1 Santa Fe Institute, Santa Fe, NM 87501, USA; 2 Department of Biology and BEACON Center for the Study of Evolution in Action, University of Washington, Seattle, WA 98195, USA; 3 Department of Biology, Georgia Institute of Technology, Atlanta, GA 30332, USA

**Keywords:** multicellularity, ratcheting, major transition, evolution, stability

## Abstract

The evolutionary transition to multicellularity probably began with the formation of simple undifferentiated cellular groups. Such groups evolve readily in diverse lineages of extant unicellular taxa, suggesting that there are few genetic barriers to this first key step. This may act as a double-edged sword: labile transitions between unicellular and multicellular states may facilitate the evolution of simple multicellularity, but reversion to a unicellular state may inhibit the evolution of increased complexity. In this paper, we examine how multicellular adaptations can act as evolutionary ‘ratchets’, limiting the potential for reversion to unicellularity. We consider a nascent multicellular lineage growing in an environment that varies between favouring multicellularity and favouring unicellularity. The first type of ratcheting mutations increase cell-level fitness in a multicellular context but are costly in a single-celled context, reducing the fitness of revertants. The second type of ratcheting mutations directly decrease the probability that a mutation will result in reversion (either as a pleiotropic consequence or via direct modification of switch rates). We show that both types of ratcheting mutations act to stabilize the multicellular state. We also identify synergistic effects between the two types of ratcheting mutations in which the presence of one creates the selective conditions favouring the other. Ratcheting mutations may play a key role in diverse evolutionary transitions in individuality, sustaining selection on the new higher-level organism by constraining evolutionary reversion.

This article is part of the themed issue ‘The major synthetic evolutionary transitions’.

## 1. Introduction

Complex life has evolved through a series of events in which organisms evolve to become specialized parts of new, ‘higher-level’ organisms [1]. These events have come to be known as major transitions in evolution [2], or evolutionary transitions in individuality [3], and include the origin of cells from groups of interacting replicators, the origin of eukaryotes from mutualistic prokaryotes, the evolution of multicellular organisms from unicellular ancestors and the evolution of eusocial ‘superorganisms’ from solitary individual multicellular organisms. The hierarchical nature of life, with genes nested within cells nested within multicellular organisms nested within societies, is a historical signature of these repeated evolutionary transitions in individuality.

Here, we focus on the evolution of multicellular organisms from unicellular ancestors. Multicellular organisms are a ubiquitous part of our environment. As Kirk [4] rightly observed ‘ … if all multicellular eukaryotes suddenly vanished from Earth, our planet would appear as barren as Mars’. Despite the profound challenges involved in making the transition, multicellularity has evolved at least 25 times in taxonomically and ecologically diverse microbial lineages [5]. Filamentous cyanobacteria are the first lineage known to evolve multicellularity on the Earth, dating between 2.25 and 2.45 billion years ago [6]. Centimetre-scale macrofossils of putative multicellular organisms composed of cells growing in radially organized sheets have also been recovered from a period of elevated oxygen 2.1 billion years ago [7], though little is known about their biology. The red algae *Bangiomorpha* is the first known multicellular eukaryote, making this transition approximately 1.2 billion years ago [8]. Within the last billion years, there have been numerous transitions to multicellularity across lineages spanning the deepest divergences within eukaryotes [9–11] and within archaea [12].

The fact that multicellularity has independently arisen so many times in diverse lineages suggests that the selective conditions favouring this transition must be rather common [5]. Theoretical and experimental works support this hypothesis, and indeed the formation of simple clusters of cells (the first step in the transition) can be adaptive under a number of distinct ecological scenarios [13]. For example, clusters may provide protection from predation [14–16] and environmental stress [17], or improved utilization of diffusible nutrients [18–20]. Experimental studies have also demonstrated that (under the right selective conditions) simple undifferentiated multicellularity evolves readily in diverse species [16,21–24], suggesting that the genetic changes necessary to achieve simple undifferentiated multicellularity are few.

Two independent experiments observed the evolution of multicellularity in the budding yeast, *Saccharomyces cerevisiae* [22,23,25]. Both found that a loss of function mutation in the transcription factor *ACE2* was enough to produce simple undifferentiated multicellularity [23,25]. In *Pseudomonas fluorescens*, another model organism for studying the evolution of multicellularity, switching between multicellular ‘wrinkly spreader’ (WS) and unicellular ‘smooth morph’ (SM) states can be achieved readily by mutations in a small number of loci that affect the production of an extracellular glue [26–28].

The evolutionary lability of multicellularity seen in experimental systems raises an interesting issue: if simple multicellularity is so easy to achieve, shouldn't it also be easy to lose? Reversion to unicellularity may, therefore, represent a significant threat to the long-term stability of multicellularity, particularly when its benefits are environmentally dependent (e.g. when predators are present). Experiments with microbes have also highlighted costs of multicellularity. In a study where selection for rapid sedimentation in liquid media promoted the evolution of multicellularity in yeast, Ratcliff *et al.* [22] found that multicellularity was associated with 10% reduced fitness in the absence of settling selection, probably due to slower growth rates caused by diffusional limitation [29]. In addition, Rainey & Rainey [27] found that the WS genotype suffered a 20% fitness cost relative to the ancestral SM genotype under conditions that did not require colonization of the air–liquid interface. Similar results have been found in natural systems. For example, the green alga *Desmodesmus subspicatus* facultatively forms multicellular colonies when it senses chemical cues released by its predator *Daphnia*, increasing fitness during predation, but in the absence of predation the unicellular phenotype displaces multicelled phenotypes [30]. This suggests that there would be strong selection for unicellular revertants from nascent multicellular organisms if the environment were to shift in such a way that groups of cells were no longer favoured. How then is multicellularity stabilized in the face of this threat?

Questions of the evolutionary stability of major transitions have long been considered of key importance [2]. Historically, evolutionary conflict between lower and higher levels of selection have been regarded as the largest threat to nascent higher-level entities [1,31,32]. During the transition to multicellularity, for example, the focus has been on explaining why selection among competing cell lineages within a single multicellular entity does not disrupt the integrity of the group. Indeed, multicellular organisms are rife with the potential for such conflict, which in animals manifests as cancer [33]. Several mechanisms that limit within-organism variation, and thus limit the potential for conflict among lower-level units, have evolved in multicellular organisms such as the early sequestration of the germ line [1] and the evolution of a single-cell bottleneck during development [34–36]. Other conflict-minimizing strategies, such as greenbeard genes [17,37] and policing [38,39], have evolved in cooperative groups that lack clonal development such as social amoebae and myxobacteria.

In this paper, we focus on how the transition to multicellularity may be stabilized against evolutionary reversion when environmental conditions change and tip the balance of selection back in favour of unicellularity. Solving this problem is necessary for the long-term success of a major transition. There are two ways that evolutionary change can limit the potential effects of reversion. The first solution we consider is for mutations that are adaptive in the multicellular context to be disadvantageous in the single-celled context. This could make reversion less beneficial and maintain selection for group cohesiveness even when the environment favours unicellularity [40,41]. Here, we refer to the accumulation of mutations that have this effect as a ‘ratcheting’ process (and traits that have this property may be referred to as ratcheting traits). Similarly, multicellularity can be stabilized if unicellularity simply becomes less accessible by mutation. This could happen via deletion of a gene essential for unicellularity or if the genetic architecture evolves in such a way that it increases the number of mutations needed to return to the unicellular state. As these processes also limit the potential effects of reversion, they can also be considered as a form of ratcheting. To delineate between the two processes, we label the accumulation of traits with different fitness characteristics in unicellular and multicellular contexts as ‘type 1 ratcheting’ and the reduction in the switch rate between unicellular and multicellular states as ‘type 2 ratcheting’.

Here, we examine both types of ratcheting and their potential to stabilize multicellularity in environments that fluctuate between selecting for unicellular and multicellular states. Through the use of mathematical models, we show that both forms of ratcheting can be effective on their own under certain conditions. Furthermore, when both types of ratcheting are permitted there are synergistic effects that increase the stability of multicellularity.

## 2. Model

We consider the evolutionary dynamics of a population of genotypes with the capacity to switch between unicellular/independent (*I*) cell types and cells that exist as part of multicellular/group states (*G* cells). While there are many modes by which multicellular groups grow and reproduce, we choose a more general, cell-level approach. We do not explicitly model a particular multicellular form or group structure. Rather, we consider the population dynamics of *I* and *G* cells where the benefit (or cost) of being multicellular manifests in the fitness values of *G* cells. So in an environment that favours multicellularity the *G* cells have higher fitness than the *I* cells. This approach eliminates the need to track which *G* cells belong to which multicellular organisms.

If there were only one environmental state then either multicellular or unicellular cell types would have a selective advantage and drive the other extinct. Instead, we assume that there is an environment that fluctuates between two states: *E_G_* and *E_I_*. The *E_I_* state favours unicellular *I* cells and the *E_G_* environmental state favours multicellular *G* cells. When exposed to either environmental state, cells reproduce until they reach a certain number, *N*, the carrying capacity (*N* = 10^5^ in this paper). Each reproductive event is chosen randomly from the current population based on the fitness values of cells. So if there is an *I* cell with fitness *k_i_* and two *G* cells with fitness *k_g_* then the probability that the *I* cell would reproduce next would be *k_i_*/(*k_i_* + 2*k_g_*). The manner in which we simulate population expansion is based on the Gillespie algorithm [42] and permits simulation of large populations with different fitness values and rare, stochastic events such as mutations. After the population reaches carrying capacity, it experiences a bottleneck, whereby a fraction of individuals (10^2^ in this paper) are chosen randomly from the population to seed growth in the next round/environmental state. Thus, populations experience cycles of expansion to 10^5^ and contraction to 10^2^.

As populations expand, reproductive events allow for chance mutations that change the fitness value of cells. At the start the fitness values are shown in table 1, where *c* is a cost of being maladapted (*c* > 0). With each reproduction there is a fixed probability *p*_f_ (10^−3^ in this paper) that a cell will gain a mutation that improves its fitness. For simplicity, we ignore deleterious mutations and consider only beneficial mutations. The maximum fitness benefit of a mutation, Δ*s*, is sampled from an exponential distribution with *λ* = 35 [43]. This is assigned to *I* cells in *E_I_* and *G* cells in *E_G_*. In addition, we assume that there is a correlation for fitness-gaining mutations such that a cell also gains a fraction of this benefit in the environment to which it is not well suited. We use a fraction of 1/5 throughout this paper. Thus, as a result of a single mutation the following fitness values can be obtained in *I* or *G* cells (table 2).

**Table 1.** Initial fitness values.

type	*E_G_*	*E_I_*
*I*	1 − *c*	1
*G*	1	1 − *c*

**Table 2.** Fitness values after a beneficial mutation.

type	*E_G_*	*E_I_*
*I*	1 − *c* + (1/5)Δ*s*	1 + Δ*s*
*G*	1 + Δ*s*	1 − *c* + (1/5)Δ*s*

At reproduction, there is also a chance that a cell can switch types between *I* and *G* cell types. This occurs randomly with probability *p_s_* and is the same for both *I* to *G* and *G* to *I* cell switching. We also assume that this probability is fixed and independent of other evolved traits including fitness-increasing mutations—later we relax part of this assumption and allow *p_s_* to evolve. If we assume that the *I* and *G* cell switch is independent of fitness-affecting mutations, it implies that the responsible mechanisms reside at different loci and have no epistatic interactions with the fitness-affecting mutations.

As a consequence of allowing the cell types to switch, we must track four fitness values: the *E_G_* and *E_I_* fitness for the current cell type and the *E_G_* and *E_I_* fitness for the opposite cell type should a switch occur. This permits the possibility that a fitness-affecting mutation in the current cell type may also affect the fitness values of the opposite cell type, which would only manifest following a switch. We consider two possibilities: coupled, contrasting fitness effects (ratcheting) or uncoupled, independent fitness effects (non-ratcheting). In the ratcheting case, a beneficial mutation in one cell type has deleterious pleiotropic effects in the opposite cell type (table 3).

**Table 3.** Fitness values after a beneficial mutation with ratcheting.

current type	*E_G_*	*E_I_*	opposite type	*E_G_*	*E_I_*
*I*	1 − *c* + (1/5)Δ*s*	1 + Δ*s*	*G*	1 − Δ*s*	1 − *c* − Δ*s*
*G*	1 + Δ*s*	1 − *c* + (1/5)Δ*s*	*I*	1 − *c* − Δ*s*	1 − Δ*s*

### (a) Results: ratcheting type 1

**Figure 1.**
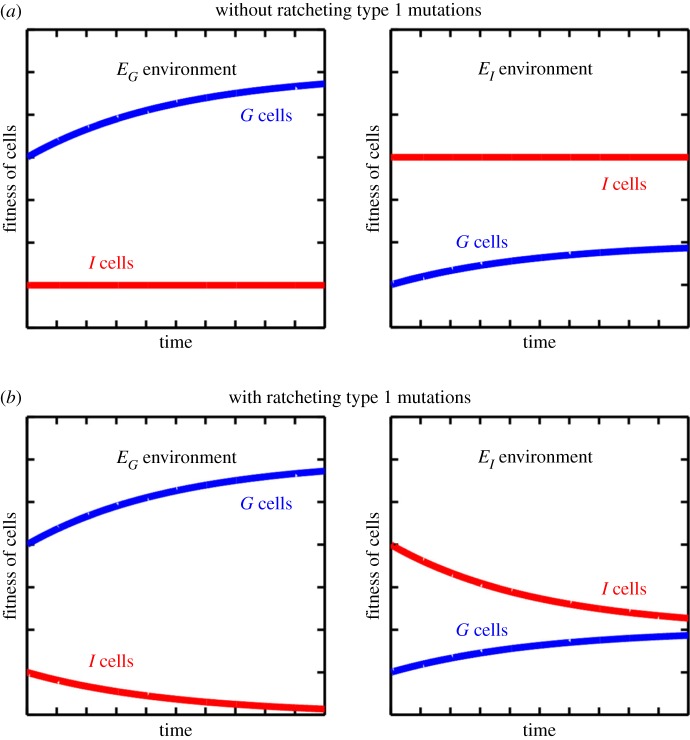
Schematic showing the effects of evolution in an *E_G_* environment on the fitness of *I* and *G* cells in environments *E_G_* and *E_I_*. (*a*) Evolution of *G* cells in an *E_G_* environment leads to increased fitness in both *E_G_* and *E_I_* environments, though the effect is smaller in *E_I_*. These fitness changes have no consequences on the fitness of *I* cells in either environment. (*b*) The addition of ratcheting effects couples increases in *G* cell fitness with decreases in *I* cell fitness in both *E_I_* and *E_G_*. Ultimately, the effect is that the relative advantage of *I* cells (derived from *G* cells by mutation) in *E_I_* is significantly decreased while the relative advantage of *G* cells in *E_G_* is increased.

The first type of ratcheting is when the accumulation of fitness-affecting traits in the multicellular context, i.e. as a *G* cell, have corresponding negative consequences in the *I* cell form. Without ratcheting, as *G* cells improve in fitness in environment *E_G_* there are no effects for the *I* cells (figure 1*a*). When compared with systems that evolve with ratcheting traits (figure 1*b*), there are two key differences: (i) the selective benefit of being a *G* cell in an *E_G_* environment increases and (ii) the selective cost of being a *G* cell in an *E_I_* environment decreases. As a result of the first effect, *G* cells progressively outcompete unicellular types driving them from the population. The second effect acts to stabilize the multicellular form because it reduces the fitness benefit of being unicellular in an *E_I_* environment. Should the environment switch from an *E_G_* state to an *E_I_* state, it will take longer for unicellular *I* cells to overtake a population of multicellular *G* cells.

**Figure 2.**
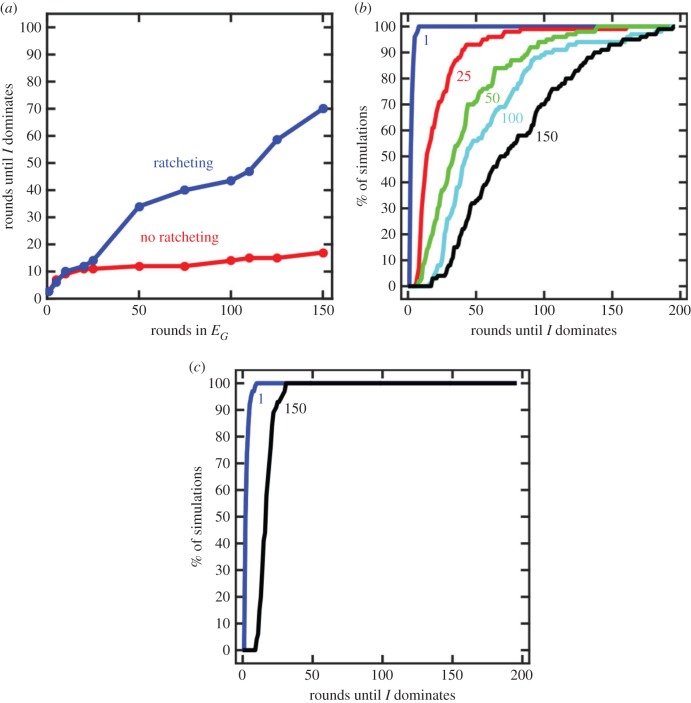
Ratcheting type 1 increases the stability of multicellularity. (*a*) The duration of *G* cells in an *E_I_* environment is shown as a function of the duration of growth in the *E_G_* environment. Each point is the median of 100 simulations. If type 1 ratcheting mutations do not occur (red) then the duration in *E_G_* has only a small effect on the stability of multicellularity by removing all pre-existing *I* cells from the population. By contrast, if ratcheting type 1 mutations occur (blue) there is a much larger increase in the stability of the multicellular form. Increased duration of growth in *E_G_* leads to increased accumulation of ratcheting traits and greater multicellular stability. (*b*) An empirical cumulative distribution function plot shows the effect of the duration of growth in *E_G_* on the variation in the persistence of multicellularity when ratcheting mutations occur. Depending on the magnitude and number of ratcheting mutations that fix in the population, the stability of multicellularity can be three to five times greater than the median. (*c*) For comparison, a similar plot is shown when there are no ratcheting mutations.

To observe the stabilizing effect of ratcheting traits, we simulated the evolution of populations grown in an *E_G_* environment for different periods of time that were then switched to an *E_I_* environment. We then determined the time it took for the *I* cell types to occupy 99% of the population (figure 2). The longer populations were exposed to the *E_G_* environment, the more ratcheting traits they accumulated and the longer it took for *I* cells to reach numerical dominance. Populations that spent too little time in *E_G_* did not accumulate enough ratcheting traits to stabilize the multicellular form. As the strength of ratcheting depends on the remaining fitness gap between *G* and *I* cells in *E_I_*, it depends on factors that influence this—such as the distribution of fitness effects for beneficial mutations and the initial fitness difference between types. If there is a larger initial gap in fitnesses between *I* and *G* cells and mutations tend to confer little advantage then it takes longer to reduce the fitness gap by a meaningful amount. In our model, if we increase the initial fitness difference from 0.1 to 0.5 then we find that the time frame and population size examined here are not enough to show a difference between evolution with and without ratcheting traits (data not shown). Inversely, an increase in the carrying capacity or the bottleneck size affords more opportunity to gain ratcheting mutations and have them fix in a population.

**Figure 3.**
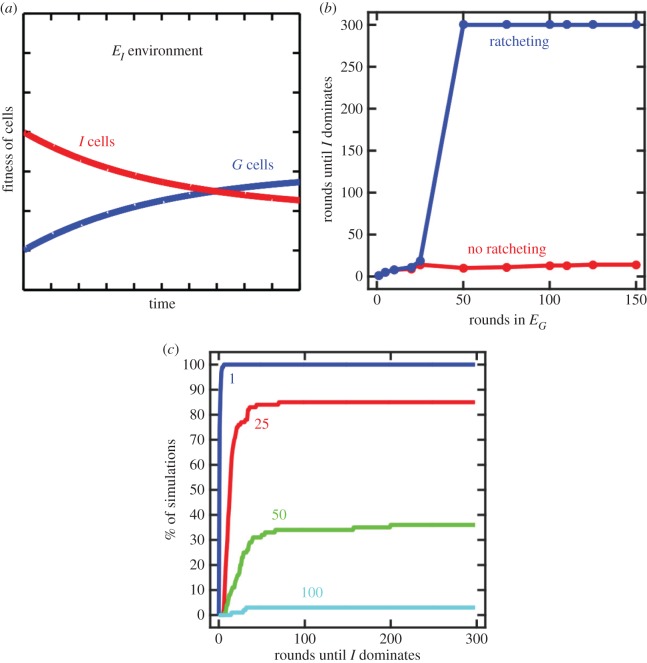
The case when *I* cells become less fit than *G* cells in the *E_I_* environment. (*a*) As a result of *G* cells evolving in an *E_G_* environment, the evolution of ratcheting traits drives the fitness of *I* cells in *E_I_* below *G* cells. (*b*) The consequence of this is that once such mutations fix, there is no selective benefit for *G* cells to revert back to *I* cells even when grown in an *E_I_* environment. The time it takes for *I* cells to occupy 99% of the population is shown by the blue curve. Each point is the median of 100 simulations. Simulations were run for only 300 rounds so a value of 300 means that *G* cells are present for the entire duration of the simulation. For comparison, the red curve shows the case without type 1 ratcheting mutations. (*c*) An empirical cumulative distribution function plot shows the variation in the stability of multicellularity for different durations of growth in *E_G_*. The value of each curve at 300 shows the percentage of simulations in which *I* cells eventually dominated the population. Those that do not reach 100 correspond to simulations in which *G* cells remained present.

A more extreme form of ratcheting can occur if *I* cells lose fitness in *E_I_* until *G* cells are fitter (figure 3). In this case, once a sufficient number of mutations have occurred there is no longer a selective advantage to producing *I* cells in any environment. Even if *G* cells were to revert to unicellular *I* cells, they would be quickly outcompeted. While this type of ratcheting might seem unlikely, it may be quite common. For example, the evolution of mutualistic interdependence among cells, a common trait in complex multicellular organisms, may result in extremely steep costs of reversion in which single cells lack the capacity to survive autonomously.

### (b) Results: ratcheting type 2

Another way that organisms can become ratcheted in a multicellular form is if the switch from *G* cells to *I* cells becomes less accessible by mutation, or if a switch is no longer possible. Such a decrease in switch rate could arise as a pleiotropic consequence of mutations that are adaptive in the multicellular context, analogous to the type 1 ratcheting case. Alternatively, when growing and evolving in an *E_G_* environment (where *G* cells have a fitness advantage), it could be independently advantageous to lower the rate of switching back to *I* cells—assuming that this trait is evolvable. To demonstrate this latter possibility, we consider a simple model with discrete time steps (equation (2.1)). During each time step, *G* cells reproduce and with probability *p* produce *I* cells. Also during the time step, a smaller fraction of *I* cells reproduce—the *c* term is the reproductive cost for being an *I* cell in an *E_G_* environment. For simplicity, we do not permit *I* cells to switch back into *G* cells—this removes higher-order terms that include the unlikely event that a cell switches between *G* and *I* forms upon every reproduction.
2.1
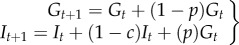


The total population of cells at time *t* can be solved analytically as follows:
2.2
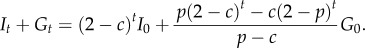


**Figure 4.**
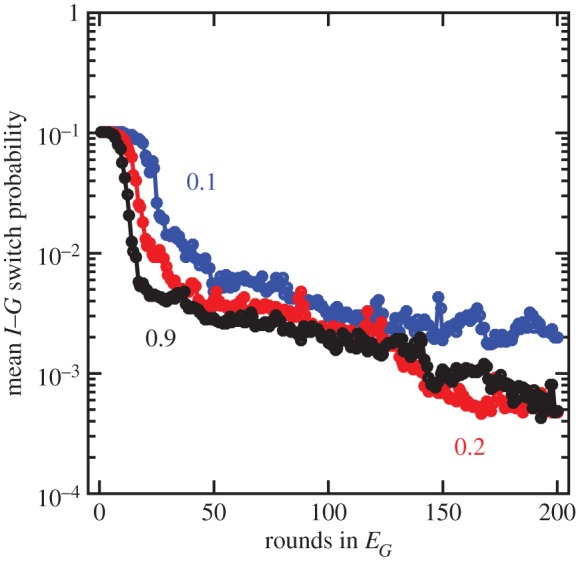
Selection for lower probability of switching. The probability of switching between *I* and *G* cells is shown as a function of the number of rounds grown in *E_G_*. Each curve is the median of 10 evolved simulations and colours correspond to different *c* values—fitness differences between *I* and *G* cells—such that blue is *c* = 0.1, red is *c* = 0.2 and black is *c* = 0.9. All populations evolve lower probabilities of switching, starting at *p* = 10^−1^ and evolving close to *p* = 10^−3^, which is the same value as the probability that a mutation changes the probability of switching.

Whenever *c* > 0, i.e. there is a cost to being an *I* cell, and equation (2.2) is a decreasing function over the range 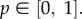 The rate of population growth is at a maximum when *p* = 0, i.e. when *G* cells stop switching to *I* cells (this is readily apparent for the extreme case of *c* = 2, where equation (2.2) reduces to *I_t_* + *G_t_* = 2(2 − *p*)*^t^*^−1^*G*_0_). Thus, with prolonged growth in the *E_G_* environment, it is advantageous for multicellular cells to decrease the rate of switching to unicellular types. We can see this in our simulation model with prolonged growth in the *E_G_* environment assuming cells can switch bidirectionally between *G* and *I* types. The average population switch rate from *G* to *I* (and vice versa) decreases with time when grown in the same environment (figure 4). Cells initially switch with probability *p* = 0.1 and evolve to switch 100-fold less frequently, at *p* = 0.001. In theory, populations could do better by switching less often than *p* = 0.001 but the relative benefit is much smaller compared with the difference between *p* = 0.1 and *p* = 0.001 and a population size of 10^5^, i.e. the benefit of slower switching declines as *p* approaches 0.

### (c) Results: combining types

Each type of ratcheting has particular conditions that make it more successful. The type 1 form of ratcheting relies on the accumulation of mutations that lower the fitness gap between *G* and *I* cells in the *E_I_* environment. As a consequence, the effectiveness of this type of ratcheting depends on the distribution of mutations and the initial gap in fitnesses that must be overcome. If there is a small fitness gap and beneficial mutations are common then type 1 ratcheting can quickly decrease the benefit of being unicellular in the *E_I_* environment. This, in turn, improves the evolutionary stability of the multicellular form should the environment switch from *E_G_* to *E_I_*. If, instead, there is a large fitness gap and beneficial mutations of sizable effect are rare then type 1 ratcheting may not be effective without prolonged time or opportunity to gain mutations in the *E_G_* environment. While large fitness gaps between *I* and *G* cells may limit the effectiveness of type 1 ratcheting, they are conducive to type 2 ratcheting. A large fitness gap imposes a significant cost on producing the maladapted phenotypes in the wrong environment and can generate selection to reduce the switch rate. By contrast, smaller fitness gaps reduce the selective pressure for type 2 ratcheting. Thus, because the two types of ratcheting are suited to different conditions, we expect that in a single selective environment one type of ratcheting will be more effective and, therefore, more likely to occur than the other.

Although the types of ratcheting are better suited to different environmental conditions, there can be a synergistic effect such that one type of ratcheting changes the selective conditions to promote the other type of ratcheting. We consider a fluctuating environment that cycles between *E_G_* and *E_I_* after a fixed period of growth in each: *n_g_* reproductive generations in *E_G_* and *n_i_* reproductive generations in *E_I_*. Equation (2.3) shows the population dynamics for growth in both environments with fitness differences *c_g_* and *c_i_* between *G* and *I* cells (*c_g_*, *c_i_* ≥ 0) in *E_G_* and *E_I_* environmental states, respectively.


2.3
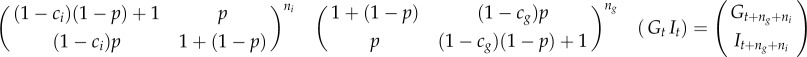


**Figure 5.**
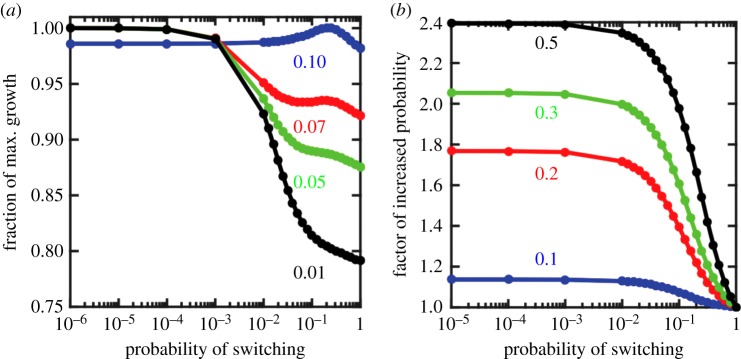
Combining ratcheting types. (*a*) Type 1 ratcheting can promote type 2 ratcheting. The fraction of maximal growth rate, as determined by the largest eigenvalues of equation (2.3), is shown as a function of the switch rate *p* for different values of *c_i_* (*c_g_* is fixed at 0.1). The blue curve shows that when *c_g_* = *c_i_* = 0.1, the optimal switch rate is *p* ≈ 0.2. When *c_g_* > *c_i_*, as a consequence of ratcheting type 1 mutations, then the optimal switch rate is *p* < 10^−6^. The red (*c_i_* = 0.07), green (*c_i_* = 0.05) and black (*c_i_* = 0.01) curves show that as the fitness asymmetry increases there is stronger selection against switching frequently. (*b*) Type 2 ratcheting can promote type 1 ratcheting. The probability of finding a beneficial mutation to overcome a fitness gap of *c* is shown as a function of the switch rate *p* for different values of *c*. Each curve represents a different fitness gap (blue is *c* = 0.1, red is *c* = 0.2, green is *c* = 0.3 and black is *c* = 0.5) and is scaled by the probability of finding a beneficial mutation when *p* = 1, i.e. the worst case scenario. Thus, the vertical axis shows the factor of improvement when switching is lowered from *p* = 1. The chance of finding a beneficial mutation to overcome *c* increases as the switch rate is lowered, which can result from type 2 ratcheting.

If the two periods are equal, *n_i_* = *n_g_* and the benefit of being *I* in *E_I_* is the same as being *G* in *E_G_* (*c_g_* = *c_i_*), then a non-zero, switch rate, *p*, maximizes growth of the collective *I* and *G* cells (see blue curve in figure 5). The exact value of the optimal switch rate depends on the particular duration in each environmental state—the longer the duration, the slower the switch rate that maximizes growth. When *n_i_*, *n_g_* = 10, there is selection for a high switch rate, close to *p* ≈ 0.2, between *I* and *G* cells. In such a case, evolution of the switch rate would not generate type 2 ratcheting. If, however, a *G* cell were to gain a type 1 ratcheting mutation that creates an asymmetry in fitness such that *c_g_* > *c_i_* then the benefit of being *G* in *E_G_* would be greater than the benefit of being *I* in *E_I_*. This fitness asymmetry creates selective pressure to lower the switch rate. Figure 5 shows that larger fitness asymmetries result in stronger selection against high rates of switching. Thus, the acquisition of type 1 ratcheting mutations can create the selective conditions that drive the evolution of type 2 ratcheting.

Alternatively, type 2 ratcheting can increase the probability of gaining type 1 ratcheting mutations. A key factor for the effectiveness of type 1 ratcheting is the time it takes to gain a mutation that can decrease the fitness gap between *G* and *I* cells in *E_I_*. Increasing the number of *G* cell reproductive events can improve the odds of finding such a mutation—especially if the fitness gap is large. To this end, type 2 ratcheting can help by decreasing the switch rate between *G* and *I* cells, and thereby giving *G* cells more reproductive opportunities to obtain a useful type 1 ratcheting mutation. If the fitness gap to overcome is *c* then the chances of getting a beneficial mutation of *c* or higher within *n* reproductive events and a mutation rate of *m* is 1 − (1 − e*^λ^*^c^)*^nm^*, where *λ* is the rate parameter for the distribution of beneficial mutations. As the fitness gap increases, the usefulness of decreasing the switch rate, i.e. type 2 ratcheting, increases (figure 5). Indeed, type 2 ratcheting can improve the odds of finding a beneficial mutation to overcome *c* = 0.5 by a factor of 2.4 and *c* = 0.75 by a factor of 3.

## 3. Discussion

A confluence of evidence suggests that simple multicellularity is relatively easy to evolve, but it is also susceptible to loss due to reversion when environmental conditions change. The simple model presented here illustrates two possible solutions to the problem of reversion (referred to here as ratcheting type 1 and type 2). In ratcheting type 1, we explore the evolution of traits that increase fitness in the multicellular context and decrease fitness in the unicellular context. As expected, with more time spent in an environment that favours multicellularity (*E_G_*) there is fixation of a greater number of ratcheting type 1 mutations. Accumulation of these mutations decreases the selective advantage of a multicellular (*G*) to unicellular (*I*) reversion mutation should the environment switch to favour unicellularity (*E_I_*). This makes it more difficult for unicellularity to re-invade and increases the chances that the multicellular form can survive until the environment switches back to favour multicellularity again. In ratcheting type 2, the reversion probability itself can evolve. With more time spent in the *E_G_* environment, there is a selective benefit to decreasing the switch rate—reducing the likelihood of a *G* to *I* reversion mutation. Although the conditions that select for each type of ratcheting are different, we found that one type of ratcheting can alter conditions to promote the other type of ratcheting and increase the stability of the multicellular state.

This work highlights the types of traits that stabilize a major evolutionary transition against reversion to a previous form/lower level. In the case of multicellularity, these traits increase fitness in the multicellular context and decrease it in the unicellular context. We speculate that traits with such an effect could be common during the early stages of a major transition because the only requirement is that they be disadvantageous outside a multicellular context. A putative example of a trait with a ratcheting effect has been identified in a yeast model of multicellularity, where selection for rapid settling in liquid media resulted in the evolution of multicellular clusters [22]. In independent replicate populations, researchers have repeatedly observed the evolution of elevated rates of apoptosis—a trait which is presumably maladaptive in the unicellular context. Mathematical modelling suggests that elevated rates of apoptosis may benefit cells in large multicellular clusters by decreasing cluster size [44]. Smaller clusters face less volumetric and nutrient flow limitations and allow populations to grow faster. In the volvocine green algae, a model system for the evolution of multicellularity with species that range from unicellular to large multicellular spherical colonies, it has been suggested that changes in the regulation of growth or in the number of successive (palintomic) cell divisions that cells undergo could be early targets for adaptation to a primitive multicellular life cycle [40,45]. If the optimal regulation of these traits for small colonial forms differs from that of the unicellular form, these traits could also behave as evolutionary ratchets. However, the distribution of ratcheting mutations is an empirical question that can only be addressed with more data. Ideally, future experimental work could assess the fitness effects of candidate ratcheting traits by performing controlled pairwise competitions where the presence of the trait of interest is manipulated in both the multicellular and unicellular context.

While ratcheting traits may act to stabilize some forms of multicellularity, there are other forms in which ratcheting traits would be detrimental. In this paper, we have been assuming that once organisms make the transition to multicellularity they no longer require a persistent unicellular form. Yet some multicellular life cycles require alternation between unicellular and multicellular life stages [46]. For instance, the slime mould *Dictyostelium discoideum* regularly switches between free-living unicellular amoeba and multicellular slugs. The multicellular form acts as a type of stress response that is triggered when resources are depleted. It allows *D. discoideum* to find new environments with abundant resources. However, *D. discoideum* cannot reap any benefits without reverting back to the unicellular form because colonization only takes place as spores that generate free-living amoeba. As a consequence of this mutual reliance on types, ratcheting into either form would be detrimental to *D. discoideum* and other organisms that rely on plasticity. It is interesting to consider whether organisms that rely on plasticity to shift between unicellular and multicellular forms have different evolutionary trajectories than those that break the plasticity to stabilize the multicellular state.

Discussions of a major transition in evolution are rarely without mention of a shift in the level of selection. Often this distinction is made in terms of MLS1 and MLS2 theory [47]: with MLS1 being used to describe the early stages of the transition where the fitness of the group is a function of the fitness of its component parts, and MLS2 applying to cases where group fitness can no longer be defined in terms of its component parts. The latter typically signifies that a successful transition has been made [48,49] and that groups themselves now exist as Darwinian individuals (i.e. they exhibit variation, heredity and differences in reproductive success [50]). One aspect of our modelling approach that has interesting implications is that we did not explicitly model multicellular groups or their reproduction. As such, the fitness of groups only acts indirectly in our model via the fitness of *G* cells, without assigning *G* cells to particular multicellular groups. This suggests that the stabilizing effects we observe due to the accumulation of ratcheting traits could apply during the early (MLS1) as well as the late (MLS2) stages of a major evolutionary transition. However, we do not provide a mechanistic explanation for how and why such traits would be favoured by natural selection.

An important limitation to our modelling approach is the lack of specificity in considering the multicellular form. We adopted a general model in which unicellular *I* and multicellular *G* cells compete in the same niche and the success of multicellularity is defined by the fitness and frequency of *G* cells without regard to how they interact with the environment or each other. Yet the benefits of multicellularity are often derived from the spatial structure of the multicellular group. For example, multicellular yeast cells are capable of growing at low density in media containing the sugar sucrose, which they break down extracellularly into monosaccharides (glucose and fructose) that can be easily imported into the cell. Without the benefit of group metabolism generating high concentrations of consumable sugars, solitary cells are unable to grow [19,23]. Not only can the spatial structure imposed by a group of cells underlie the benefit of multicellularity, it can influence the evolution of novel traits [25,44,51] and may play a role in determining the likelihood of reversion. Structures that impose reproductive division of labour or physical barriers to cells abandoning groups may inhibit reversion and stabilize multicellularity. By contrast, more flexible spatial structures such as the wrinkly mats in the *P. fluorescens* experimental system permit frequent reversions to unicellularity [26–28]. Although the specific reasons that multicellular groups benefit in the *E_G_* environment will depend on the details of the system under study, our models do not consider the causal link between multicellular form and fitness. Instead, we identify general conditions under which mutations limiting evolutionary reversion to unicellularity can evolve.

In this paper, we explore how adaptations that limit the potential effects of evolutionary reversion may stabilize nascent major evolutionary transitions. Using the evolution of multicellularity from unicellular ancestors as an example, we allowed for two types of mutations to occur in our model—mutations that are beneficial in the multicellular context but deleterious in the unicellular context and mutations that affect the rate at which cells switch from the multicellular to the unicellular state. The evolution of these ratcheting traits may also play a key role in facilitating the evolution of increased complexity. By limiting the rate that unicellular revertants are produced (type 2) and the benefit of reversion (type 1), ratcheting mutations ensure that selection has sufficient time to act in the higher-level context, allowing lineages in the early stages of a major evolutionary transition opportunities to evolve increased complexity (functional integration, division of labour, etc.) via the gradual accumulation of novel traits that improve fitness in this higher-level context. By stabilizing the earliest steps in an evolutionary transition in individuality, ratcheting traits may provide a simple and robust stepping stone on the path towards increased biological complexity.

## Supplementary Material

Computer code functions

## Supplementary Material

Computer code script

## Data accessibility

Sample computer code is provided as the electronic supplementary material. Additional data and code are available upon request.
